# Multiple micronutrient supplementation for maternal anemia prevention (MMS-MAP): an individually randomized trial of higher-dose iron (60 mg, 45 mg) compared to low-dose iron (30 mg) in multiple micronutrient supplements in pregnancy

**DOI:** 10.1186/s13063-025-08906-7

**Published:** 2025-06-14

**Authors:** Emily R. Smith, Alfa Muhihi, Blair J. Wylie, Sabina Mugusi, Said Aboud, Mohamed Bakari, Wafaie Fawzi, Shabani Kinyogoli, Erin M. Oakley, Qing Pan, Mary M. Sando, Victoria S. Brownlee, Andrea B. Pembe, Christopher R. Sudfeld, Honorati Masanja

**Affiliations:** 1 Department of Global Health, The Milken Institute School of Public Health, The George Washington University, Washington, D.C. 20053 USA; 2 Africa Academy for Public Health, Dar Es Salaam, Tanzania; 3 Columbia University Medical School, Columbia University, New York, NY 10032 USA; 4 Department of Clinical Pharmacology, Muhimbili University of Health and Allied Sciences, Dar Es Salaam, Tanzania; 5 Department of Microbiology and Immunology, Muhimbili University of Health and Allied Sciences, Dar Es Salaam, Tanzania; 6 National Institute for Medical Research, Dar Es Salaam, Tanzania; 7 Department of Pediatrics and Child Health, Muhimbili University of Health and Allied Sciences, Dar Es Salaam, Tanzania; 8 Harvard University T.H. Chan School of Public Health, Harvard University, Boston, MA 02115 USA; 9 Department of Statistics, Columbian College of Arts & Science, The George Washington University, Washington, D.C. 20053 USA; 10 Department of Obstetrics and Gynaecology, Muhimbili University of Health and Allied Sciences, Dar Es Salaam, Tanzania; 11 Ifakara Health Institute, Dar Es Salaam, Tanzania

**Keywords:** Multiple micronutrient supplements, Dietary supplements, Pregnancy, Pregnancy complications, Anemia, Iron, Iron deficiency, Randomized trial

## Abstract

**Background:**

Antenatal multiple micronutrient supplementation (MMS) has been shown to be more effective than iron-folic acid (IFA) alone in reducing adverse pregnancy and birth outcomes. However, there is a concern that MMS containing 30 mg of iron may be less effective in reducing maternal anemia compared to IFA supplements containing 60 mg of iron. This poses a clinical and programmatic dilemma for countries with a high burden of maternal anemia (> 40% prevalence) where the World Health Organization (WHO) recommends using IFA with 60 mg of iron.

**Methods/design:**

We will conduct an individually randomized, quadruple-blind superiority trial of daily antenatal MMS in Dar es Salaam, Tanzania (*n* = 6381 pregnant women). Participants will be randomized to receive a daily MMS regimen during pregnancy containing 60 mg iron, 45 mg iron, or 30 mg iron at a ratio of 1:1:1. The trial participants, outcome assessors (research staff and care providers), investigators, trial statistician, and data analysts will be blinded. Pregnant women will be enrolled in the trial before 20 weeks of gestation and will receive the randomized MMS regimen from enrollment until the time of pregnancy outcome/delivery.

The primary outcome is maternal third-trimester moderate or severe anemia (Hb < 10.0 g/dL). The proportion of women who have moderate or severe anemia at 32 weeks of gestation will be compared between MMS containing 60 mg iron versus MMS containing 30 mg iron, as well as MMS containing 45 mg iron versus MMS containing 30 mg iron. Secondary outcomes include maternal hemoglobin concentration, anemia, maternal iron deficiency, and maternal iron deficiency anemia at 32 weeks gestation and 6 weeks postpartum; preeclampsia, antepartum bleeding, postpartum hemorrhage, maternal peripartum infection, pregnancy-related death, symptoms consistent with depression, fatigue, and maternal malaria during pregnancy and 42 days following; fetal death, stillbirth, birth weight, low birthweight, gestational age at birth, preterm birth, birthweight for gestational age, and small-for-gestational age birth; infant hemoglobin concentrations, infant iron status, neonatal death, and infant death at 6 weeks of age; and maternal side effects. Relative risks for binomial outcomes and mean differences for continuous outcomes and their 95% confidence intervals will be calculated for all the primary and secondary outcomes.

**Discussion:**

This study will produce causal evidence on whether MMS containing 60 or 45 mg of iron is superior to MMS containing 30 mg of iron in reducing maternal anemia and improving other important maternal and infant health outcomes. The findings of this study will inform Tanzania and similar contexts on the optimal formulation of MMS as many countries begin transitioning from IFA to MMS.

**Trial registration:**

ClinicalTrials.gov NCT06079918. Registered on 2023–10-06.

**Trial status:**

The trial is recruiting. We report protocol version 1.7 dated March 2, 2025. Recruitment started with the first patient enrolled on March 3, 2025. At the submission of this manuscript on April 10, 2025, 111 participants have been randomized. Recruitment is ongoing and should be completed by December 2026.

**Supplementary Information:**

The online version contains supplementary material available at 10.1186/s13063-025-08906-7.

## Background

Globally, about one-third of women of reproductive age, or about 613 million, are estimated to be anemic [1]. The prevalence of anemia is estimated to be highest in low- and middle-income countries (LMICs), with especially high levels in South Asia and sub-Saharan Africa. At the population level, anemia can be classified as a moderate (20–39% prevalence) or severe (≥ 40% prevalence) public health problem [2]. The 2022 Demographic and Health Survey (DHS) in Tanzania determined that the prevalence of anemia (hemoglobin < 11 g/dL) among pregnant women in the country was 56% [3]. There has been minimal change in the prevalence of anemia between the 2005, 2015, and 2022 Tanzania DHS [3–5]. Therefore, anemia among pregnant women remains a persistent severe public health problem in Tanzania.

It is estimated that about half of the anemia cases worldwide are attributed to iron deficiency, while other causes include parasitic diseases such as malaria, hookworm infections, and schistosomiasis; other micronutrient deficiencies including folic acid, vitamin A, and vitamin B12; and red blood cell conditions including sickle cell disease and thalassemia [6–8]. It is well documented that low hemoglobin in pregnancy is associated with adverse maternal and neonatal health outcomes; a meta-analysis of 95 studies found maternal hemoglobin concentrations < 11 g/dL were associated with increased risk of postpartum hemorrhage, preeclampsia, preterm birth, low birth weight, small-for-gestational-age, stillbirth, postpartum hemorrhage, neonatal death, and perinatal death [9]. The World Health Organization (WHO) currently recommends routine antenatal iron-folic acid (IFA) supplementation containing 30–60 mg of elemental iron to prevent maternal anemia and also states that 60 mg of iron is preferred in contexts where anemia is a severe public health problem [10]. The most recent Cochrane Review, which included evidence from randomized controlled trials, found that iron supplementation (including a range of doses from 20 mg to more than 200 mg per day) in pregnancy reduced maternal anemia at term by 70% and there was some evidence that iron may reduce the risk of low birth weight (11 trials; iron dose range 20–60 mg; RR 0.84; 95% CI 0.69 to 1.03) and preterm birth (13 trials; iron dose range 20–200 mg; RR 0.93; 95% CI 0.84 to 1.03) compared to controls [11]. Further, a meta-analysis of randomized trials found that iron supplementation (with doses ranging from 10 to 900 mg daily) increased hemoglobin concentration by 4.59 g/L (95% CI 3.72 to 5.46) compared with placebo or control and significantly reduced the risk of low birth weight (RR 0.81; 95% CI 0.71 to 0.93) [12].

Evidence from randomized trials also suggests there is a dose–response relationship between iron dose and maternal and fetal outcomes. The 2015 Cochrane Review of iron supplements versus placebo suggested that a low daily dose (≤ 30 mg) likely reduced maternal anemia at term by 51% based on 3 trials, while a single trial in the medium daily dose category (31–59 mg) reduced the risk by 79%, and 10 trials in the higher daily dose group (≥ 60 mg) suggested a 75% reduction [11]. Furthermore, an exposure–response analysis of randomized trials estimated that for every 10 mg increase in iron dose/day up to 66 mg/day, the relative risk of maternal anemia was 0.88 (0.84 to 0.92) [12]. On the other hand, individuals have also advocated for a lower dose of iron during pregnancy due to concerns about side effects or adverse effects of excess iron. Side effects of iron supplementation during pregnancy include nausea, dizziness, abdominal discomfort, diarrhea, constipation, and headaches, which may increase with dose [13]. There is also some evidence that iron supplementation among iron-sufficient individuals may increase the risk of infections, preeclampsia, prematurity, and fetal growth restriction [14]. The European Food Safety Authority (EFSA) released *Scientific opinion on the tolerable upper intake level for iron* in June 2024 and noted there was insufficient evidence to establish a tolerable upper intake level, but instead set a safe level of total iron intake at 40 mg/day for pregnant and lactating women [15]. There is no clarity on the optimal dose of supplemental iron that should be used in pregnancy in settings such as Tanzania, where anemia is a public health problem.

In addition to iron deficiency, pregnant women in LMICs are also at risk of multiple other micronutrient deficiencies due to inadequate dietary intake and limited diet diversity [16]. Deficiencies in other micronutrients, including vitamins A, B-complex, D, E, zinc, calcium, copper, magnesium, selenium, and iodine, are also prevalent in low-income and middle-income countries and can lead to poor pregnancy, fetal growth, and child health outcomes [17]. As a result, maternal multiple micronutrient supplementation (MMS), or multivitamins, including iron and folic acid, is a potential intervention to improve maternal and child health as compared with iIFA supplementation alone. The most recent Cochrane review found that MMS including iron and folic acid reduced the risk of low birth weight and small-for-gestational age (SGA) as compared to IFA supplementation alone [18].

The 2020 WHO antenatal care guidelines state that MMS is recommended in the context of rigorous research [19], and the guidelines note “…more evidence is needed on the effects of switching to a 30 mg dose of iron from a higher dose of iron (e.g. 60 mg), particularly in settings where higher doses of iron are routinely used due to a high anemia prevalence or other reasons” [19]. This statement stems from the fact that daily IFA supplementation with 60 mg iron is preferred in the context where anemia among pregnant women is a severe public health problem [10] (i.e., at least 40% of pregnant women have a hemoglobin level below 11 g/dL), but the standard United Nations International Multiple Micronutrient Antenatal Preparation (UNIMMAP) MMS formulation containing 15 vitamins and minerals includes only 30 mg of iron (Table 1). Thus, there is a concern, particularly in contexts with a high anemia burden where IFA containing 60 mg of iron is currently used, that switching to MMS with 30 mg of iron may increase the risk of maternal anemia.

**Table 1:** Standard UNIMMAP formulation for MMS

Micronutrient	Dose
Vitamin A	800 µg
Vitamin D	5 µg
Vitamin C	70 mg
Vitamin E	10 mg
Vitamin B1	1.4 mg
Vitamin B2	1.4 mg
Vitamin B3 (niacin)	18 mg
Vitamin B6	1.9 mg
Folic acid	400 µg
Vitamin B12	2.6 µg
Iron	30 mg
Iodine	150 µg
Zinc	15 mg
Selenium	65 µg
Copper	2 mg

This individually randomized, quadruple-blind, parallel-group superiority trial will assess the effect of MMS containing 60 and 45 mg of iron as compared to MMS containing 30 mg of iron (standard UNIMMAP formulation) on maternal anemia and other important maternal and infant health outcomes.

## Methods

### Study design

We will conduct an individually randomized, quadruple-blind (blinded trial participants, investigators, data collectors and data analysts), superiority trial of daily antenatal MMS supplementation containing 60 mg elemental iron or 45 mg elemental iron as compared to MMS containing 30 mg elemental iron. This trial protocol was written in accordance with the Standard Protocol Items: Recommendations for Interventional Trials (SPIRIT) checklist (see Additional file 1).

### Study setting

In Tanzania, an estimated 56% of pregnant women are anemic based on the 2022 Demographic and Health Survey [3]. Two previous trials of MMS versus IFA, in which both groups received 60 mg iron, have been conducted in the country, and both indicated the beneficial effects of MMS [20, 21]. Tanzania is currently considering switching to MMS as the standard of care, but there are concerns about reducing the iron dose from 60 mg in the current IFA to 30 mg in MMS. The study will be conducted at selected public antenatal care clinics in Dar es Salaam, Tanzania. The study clinics currently provide all pregnant women with IFA containing 60 mg iron free of charge as the standard of care. The same study clinics were part of a recent non-inferiority trial of low-dose calcium supplementation in pregnant women [22].

### Eligibility criteria and recruitment

**Fig. 1:**
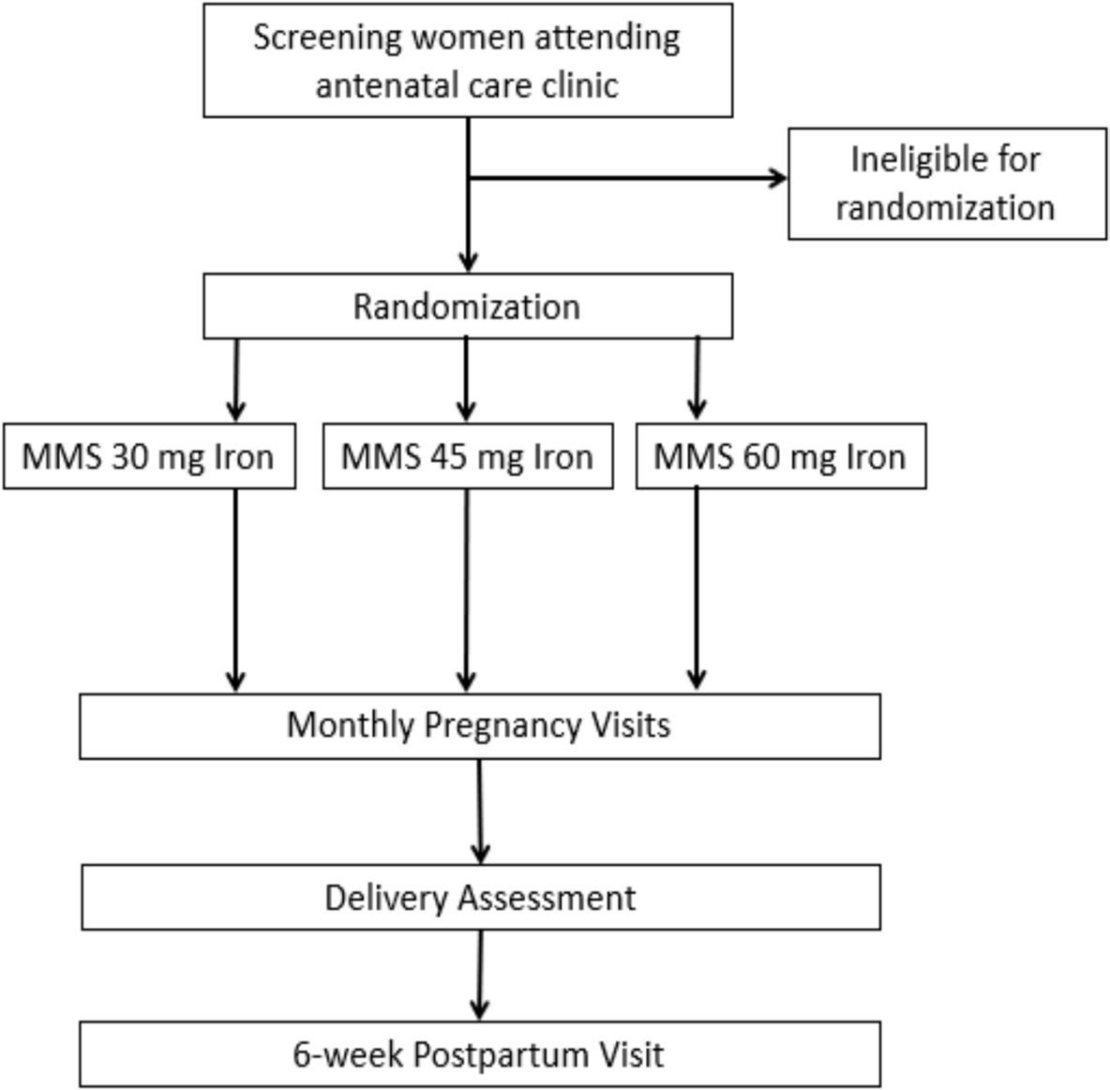
MMS-MAP trial flow diagram

The trial flow diagram is shown in Fig. 1. Research staff will assess the eligibility criteria for pregnant women who present for antenatal care at study clinics. The trial inclusion criteria are (i) pregnant women attending their first antenatal care visit, (ii) less than 20 weeks of gestation based on the last menstrual period (LMP), (iii) adult ≥ 18 years old, (iv) intending to stay in the study area until 6 weeks postpartum, and (v) provide written informed consent. Trial exclusion criteria are (i) severe anemia defined as a hemoglobin concentration < 8.5 g/dL per Tanzania standard of care; (ii) sickle cell disease including genotypes HbSS, HbSC or hemoglobin C disease (HbCC); and/or (iii) concurrent enrollment in another nutritional clinical trial. At the screening visit, participants who consent will have a finger prick for the collection of capillary blood that will be used to quantify hemoglobin concentration with the point-of-care HemoCue Hb 301 system (HemoCue AB, Ängelholm, Sweden). The HemoTypeSC test (Silver Lake Research Cooperation, Irwindale, USA) will be used for point-of-care hemoglobinopathy screening. Participants will also be asked to provide written consent for the storage of their data and blood specimens in future studies.

### Interventions

Pregnant women will be randomized to one of three trial regimens (i) MMS that contains 15 micronutrients, including 30 mg elemental iron (standard UNIMMAP formulation), (ii) MMS that contains 45 mg elemental iron plus the standard UNIMMAP formulation for the other 14 micronutrients, or (iii) MMS that contains 60 mg elemental iron plus the standard UNIMMAP formulation for the other 14 micronutrients. The only difference between the randomized groups will be the amount of iron contained in the MMS supplements. Iron will be given in the form of ferrous sulfate for all three groups. To ensure blinding, the three MMS supplements will have the same appearance, color, odor, taste, size, and weight. The trial regimen will be manufactured by DSM Nutritional Products South Africa (Gauteng, South Africa).

All participants will be counseled to take one MMS (with IFA) tablet orally per day from randomization until delivery. Participants will receive a 35-day supply of MMS in blister packs at the randomization visit and at each subsequent monthly pregnancy visit (Additional file 2). Multiple strategies will be used to enhance participant adherence to the MMS regimens. At each pregnancy visit, research staff will take a pill count of tablets returned in the regimen blister packs. Study staff will then counsel participants on MMS and provide advice on overcoming side effects and other potential barriers. Text message reminders for adherence and upcoming study visits will also be sent to study participants. Participants who report they plan to travel outside of the study area may be given extra blister packs of the regimen to ensure daily regimen supply.

### Concomitant care provided during the trial

At each trial site clinic, all participants will be provided with the standard of care throughout the study according to the national guidelines for antenatal care in Tanzania. Participants will have access to study clinics for post-trial care through the routine health system, and in the case diagnoses such as hypertensive disorders of pregnancy, red blood cell disorders, or depression are diagnosed through study procedures, we will refer participants to appropriate standard care. Any woman who is identified to be severely anemic (Hb < 8.5 g/dL) during the study will be discontinued from taking study supplements and referred for treatment according to the Tanzanian antenatal guideline for the management of severe anemia in pregnancy. We will continue to follow the women with severe anemia per the standard visit schedule according to the protocol, but they will not receive the trial regimen.

### Assignment of interventions: allocation and blinding

Pregnant women will be randomized in a 1:1:1 ratio to the three trial groups. The allocation sequence will be generated by two non-study staff at George Washington University through computer-generated randomization lists that will be stratified by the study clinic and block randomization (block sizes of 9). The two non-study staff hold the randomization list codes until completion of the primary trial analysis or as requested by the Data and Safety Monitoring Board (DSMB). An independent study pharmacist will privately prepare regimen blister packs with participant IDs based on the randomization lists for each clinic. At the randomization visit, research staff will assign pregnant women to the next available participant ID, which corresponds to a set of pre-labeled blister packs. The trial statistician and data analysts will be blinded to the treatment allocation for the primary statistical analysis. Therefore, the trial is quadruple blind because all trial participants, investigators, outcome assessors, and trial data analysts will be unable to determine the randomized group for any individual participant and will not be able to determine participants who are in the same randomized group. Furthermore, the randomization procedures will ensure complete allocation concealment.

### Sample size

We will perform three statistical tests of superiority for the primary outcome: (i) MMS 60 mg iron versus MMS 30 mg iron; (ii) MMS 45 mg iron versus MMS 30 mg iron; (iii) MMS 60 mg iron versus MMS 45 mg iron. Therefore, to account for multiple testing, the α was set to 0.01667 (0.05/3). Table 2 presents a summary of the outcome prevalence assumptions, relative risks, and power calculations for the trial. Based on data from a prior calcium trial conducted in Tanzania, we expect the prevalence of the primary outcome of maternal third-trimester moderate or severe anemia to be 25% in the MMS 45 mg iron group. A meta-analysis of iron trials and cohort studies suggests that there is a 0.88 relative risk for anemia per 10 mg increase in iron dose [12]. Therefore, we assumed a relative risk of 0.82 for comparisons with a 15 mg difference in iron (MMS 45 mg iron vs MMS 30 mg iron and MMS 60 mg iron vs 45 mg iron) and a relative risk of 0.67 for the comparison with a difference of 30 mg iron (MMS 60 mg iron versus MMS 30 mg iron). Based on these assumptions, we require third-trimester hemoglobin data from 1,808 women per arm. Assuming a conservative 15% of pregnant women will have missing third-trimester hemoglobin data (including 5% fetal loss and 10% missing blood samples), the total sample size for the trial will be 6381 (2,127 participants in each of the three randomization groups). R pwr package version 1.3 was used for sample size calculations [23].

**Table 2:** Power assuming *n* = 2,127 pregnant women randomized in each group with primary endpoint data (third-trimester anemia) available for 1,808 in each group

Comparison	Prevalence in treated	Prevalence in comparison	Assumed RR	Power
60 vs 30 mg	20.5%	30.5%	0.67	> 99%
45 vs 30 mg	25%	30.5%	0.82	90%
60 vs 45 mg	20.5%	25%	0.82	80%

### Participant timeline

**Fig. 2:**
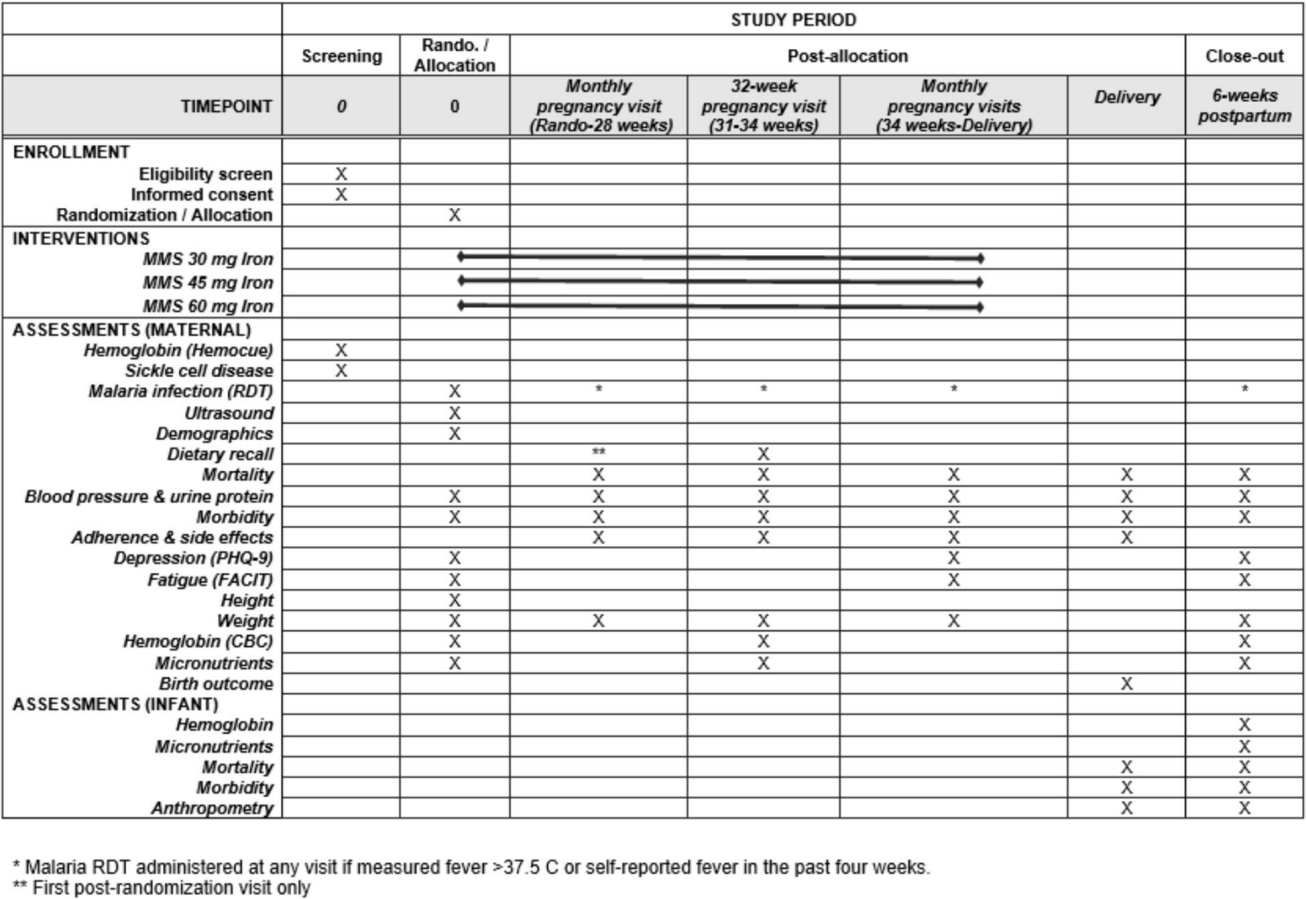
Schedule of enrollment, interventions, and assessments (SPIRIT figure)

An overview of screening, randomization, and follow-up is presented in Fig. 2. Study visits include a randomization visit and pregnancy study visits every 4 weeks until delivery. Regardless of pregnancy outcome, we will also conduct a visit around the end of the pregnancy and a postnatal visit scheduled after 42 days postpartum. Participant retention will be promoted at clinic visits and through phone calls and home visits.

### Pregnancy study visits

Pregnant participants will have a study visit at the time of randomization and monthly follow-up visits until delivery. Starting at 32 weeks of gestation, all participants will also receive weekly phone calls to check their pregnancy status. Participants will be asked to contact the research team at the time of labor onset. All women will receive an ultrasound assessment of fetal biometry around the time of randomization. All ultrasound images will be electronically stored, and a random 10% will be assessed for quality by the study obstetrician and the ultrasound quality control team. Participants will also receive a malaria rapid diagnostic test (RDT) at the first antenatal care visit, the time of randomization, as the standard of care. At pregnancy follow-up visits, participants who report a history of fever during the last month or have a body temperature of > 37.5 ℃ measured at the study visit will also have a malaria RDT performed. At all pregnancy visits, study physicians will perform a clinical exam and treat all comorbidities per Tanzanian standard of care. Blood pressure will be assessed with electronic blood pressure monitors and standardized procedures. Participants will also provide urine samples at each pregnancy visit, and dipsticks will be used to assess proteinuria. Nurses will measure maternal height with stadiometers at the randomization visit. Maternal weight will be taken on digital weighing scales, and the mid-upper arm circumference (MUAC) will be taken with a measuring tape at randomization and each pregnancy visit. A 24-h diet recall will be conducted at the first pregnancy follow-up visit and at 32 weeks of gestation. The Patient Health Questionnaire (PHQ)−9 and FACIT Fatigue Scale [24, 25] will be administered to assess symptoms of depression and symptoms of anxiety and fatigue, respectively, at randomization and at 32 weeks of gestation.

### Labor and delivery study visit

Clinic records and interviews with the clinic staff and participants will be used to ascertain labor and delivery information. Participants who deliver outside of the study area will be reached by phone by the research staff to obtain relevant information from the mother and/or facility records. Maternal blood pressure and proteinuria will be assessed by clinic or study staff or collected from clinic records. Infant length and weight will also be taken by study staff or recorded from facility records.

### Postpartum study visit

Women or women/infant pairs will have a study visit at 6 weeks postpartum (42 days) and will be discharged from the trial after completion of this visit. At the postpartum visit, research staff will assess maternal and infant morbidity and hospitalization history. Nurses will also collect weight and blood pressure from mothers. Study nurses will assess infant feeding, infant weight with a digital infant balance scale to the nearest gram, and infant length to 1-mm precision with a rigid length board with an adjustable footpiece. Study nurses will measure infant head circumference and MUAC with a flexible measuring tape. All infant anthropometric measurements will be recorded in duplicates. A verbal autopsy will be conducted to ascertain the cause of infant death [21].

### Data management

All data will be entered into an electronic data capture system developed in Tanzania, and the program will have in-built skip patterns and range check validations for each variable. All identifiable electronic data will remain in Tanzania and will be stored on secure local servers that are accessible only by the respective study data teams and investigators. Data from the trial will be stored indefinitely on servers in Tanzania and the USA.

## Outcomes

The primary outcome for the trial is the proportion of maternal participants with moderate or severe anemia, defined as hemoglobin level < 10 g/dL, during the third trimester at the 32-week visit. Hemoglobin will be measured by a hematology autoanalyzer at a central lab. The lab will maintain enrollment in an external quality assurance program to ensure consistent measurement.

The secondary outcomes related to maternal health, birth outcomes, and infant health are listed in Table 3. A maternal morbidity secondary outcomes committee composed of the trial investigators and led by a maternal–fetal medicine physician (BJW) will review all potential events related to maternal morbidities (preeclampsia, hemorrhage, and infection), blinded to the randomized treatment group using established criteria. For birth outcomes related to gestational age at birth, we will use the best obstetric estimate (BOE) approach for gestational age dating. The BOE combines two information sources: the date of the last menstrual period (LMP) and the first fetal ultrasound assessment obtained. The BOE is determined by comparing the LMP and ultrasound gestational age assessment and selecting the most probable age based on the American College of Obstetrics and Gynecology algorithm [26]. In the case that an ultrasound scan could not be done or is assessed to be invalid, we will use the LMP-based gestational age.

**Table 3:** Secondary outcome definitions

	**Definition**	**Time period(s)**
Maternal health outcomes		
Hemoglobin concentration	Continuous hemoglobin (Hb) concentration measured from venous blood by complete blood count	1. 3rd trimester 2. 6 weeks postpartum
Proportion of women with anemia (mild, moderate, or severe)	Hb concentration < 11 g/dL	3rd trimester
Proportion of women with postpartum anemia (mild, moderate, or severe)	Hb concentration < 12 g/dL	6 weeks postpartum
Maternal iron status	Continuous inflammation-adjusted serum ferritin^1^ and serum transferrin receptor	3rd trimester
Proportion of women with iron deficiency	Iron deficiency: inflammation-adjusted serum ferritin^1^ < 15 µg/L	3rd trimester
Proportion of women with iron deficiency anemia	Anemia (Hb < 11 g/dL) and iron deficiency (inflammation-adjusted serum ferritin^1^ < 15 µg/L)	3rd trimester
Proportion of women with preeclampsia	Gestational hypertension and gestational proteinuria among participants without chronic hypertension or gestational proteinuria among participants with chronic hypertension (superimposed preeclampsia) or clinical diagnosis of preeclampsia by managing clinical team or development of severe features of preeclampsia even in the absence of proteinuria	20 weeks gestation to delivery
Proportion of women with antepartum bleeding	Self-reported or clinical diagnosis of bleeding from or into the genital tract	From 24 weeks gestation through delivery
Proportion of women with postpartum hemorrhage	Clinical diagnosis of postpartum hemorrhage or use of critical interventions to treat PPH	From delivery through 42 days post delivery
Proportion of women with maternal peripartum infection	Categories of infection are defined based on severity: 1. Infection-related severe maternal outcomes: hospitalization for infection, women presenting with WHO near-miss criteria to define organ system dysfunction, invasive procedure to treat the source of infection (vacuum aspiration, dilatation and curettage, wound debridement, drainage, laparotomy and lavage, other surgery), or maternal death 2. Less severe infections: all other incident infections	Pregnancy, labor, delivery, and up to 42 days postpartum
Proportion of women with pregnancy-related death	Death of a woman while pregnant or within 42 days of termination of pregnancy, irrespective of the cause of death	During pregnancy or within 42 days of termination of pregnancy
Proportion of women with symptoms consistent with perinatal depression	Based on the PHQ-9 screening checklist score [24]	3rd trimester
Proportion of women with symptoms consistent with postpartum depression	Based on the PHQ-9 screening checklist score [24]	6 weeks postpartum
Proportion of women with symptoms consistent with fatigue	Based on the FACIT assessment for fatigue [25]	1. 3rd trimester 2. 6 weeks postpartum
Proportion of women with malaria infection	Based on HRP2 biomarker (indicative of current or recent malaria infection) or rapid diagnostic test	During pregnancy, labor or within 42 days of termination of pregnancy
Birth outcomes		
Proportion of fetal death	A product of human conception, irrespective of the duration of the pregnancy, which, after expulsion or extraction, does *not* breath or show any other evidence of life such as beating of the heart, pulsation of the umbilical cord, or definite movement of voluntary muscles, whether or not the umbilical cord has been cut or the placenta is attached	Pregnancy termination
Proportion of stillbirth	Fetal death ≥ 28 weeks gestation [27]	Pregnancy termination
Birthweight	Continuous birthweight among live births	Birth
Proportion of low birthweight	Live birth with birthweight < 2500 g	Birth
Gestational age at birth	Duration of gestation in weeks as a continuous measure among live births based on the best obstetric estimate	Birth
Proportion of preterm birth	Live birth < 37 weeks gestation (based on best obstetric estimate)	Birth
Birthweight for gestational age	Continuous centile based on INTERGROWTH-21st standard birth centile among live births [28]	Birth
Proportion of small-for-gestational age birth < 10th percentile	Size-for-gestational age < 10th percentile on the INTERGROWTH-21st standard among live births [28]	Birth
Proportion of small-for-gestational age birth < 3rd percentile	Size-for-gestational age < 3rd percentile on the INTERGROWTH-21st standard among live births [28]	Birth
Infant outcome		
Infant hemoglobin concentration	Continuous Hb concentration measured by HemoCue Hb 301	6 weeks of age
Infant iron status	Continuous inflammation-adjusted serum ferritin^1^	6 weeks of age
Proportion of infants with iron deficiency	Iron deficiency: inflammation-adjusted serum ferritin^1^ as < 20 µg/L	6 weeks of age
Proportion of neonatal death	Death of liveborn infant during the first 28 days of life	Birth to 28 days of age
Proportion of infant death < 42 days	Death of a live born infant during the first 42 days of life	Birth to 42 days of age

^1^We will adjust serum ferritin and sTfR using the Biomarkers Reflecting Inflammation and Nutritional Determinants of Anemia (BRINDA) approach by accounting for alpha-1-glycoprotein 1 (AGP) and C-reactive protein (CRP)

The following outcomes will be included in the analyses of side effects and adherence to the MMS regimens. These are consistent with the side effects reported in the WHO 2016 Guidelines for a Positive Pregnancy Experience for iron and folic acid supplementation [10] (Table 4).

**Table 4:** Adherence and side effect outcome definitions

Outcome	Definition	Timeframe
Percent adherence	The percentage of days a pregnant woman took an MMS pill out of the total number of days from randomization to delivery	Duration of pregnancy
Proportion reporting any side effect	Reported any of the symptoms listed below during the intervention period	Duration of pregnancy
Diarrhea	● Side effects self-reported within the past 4 weeks ● Number of days the symptoms were experienced in the past 4 weeks (since the last visit)	Duration of Pregnancy
Heartburn		
Constipation		
Vomiting		
Nausea		
Leg cramps		
Low back/pelvic pain		

## Statistical methods

An intent-to-treat (ITT) approach (as-randomized) with a complete case analysis (among participants with outcome data) will be the primary analytic strategy for all primary and secondary outcomes. The ITT analysis for the primary outcome of maternal moderate or severe anemia will include all women with data on 3rd-trimester hemoglobin concentrations. All participant data collected until completion of the study, withdrawal, or loss to follow-up will be used in the analysis, except in the case that the study participant requests their data or blood samples be destroyed. Log-binomial models, including a fixed effect for study clinic to account for stratified randomization [29], will estimate the relative risks, 95% confidence intervals, and *p*-values for the difference in moderate and severe anemia for the three primary comparisons (60 mg vs 30 mg, 60 mg vs 45 mg and 45 mg vs 30 mg).

We will also evaluate the effect of the randomized regimens on secondary outcomes defined in Table 3. For binomial secondary endpoints, we will use similar methods to the primary analysis, while continuous secondary outcomes will use linear regression models to estimate mean differences. For infant outcomes, mixed effects models will be used for both binomial and continuous outcomes to account for correlation due to multiple gestations (e.g., twins).

## Oversight and monitoring

The trial team will seek ongoing support from a Technical Advisory Group (TAG) of experts in micronutrient supplementation in pregnancy, randomized trials, and obstetrics. The TAG will meet with the study team annually to provide guidance on the implementation of the trial, troubleshoot issues as they may arise, and contribute to the dissemination activities of the trial. The trials will be overseen by a Data and Safety Monitoring Board (DSMB) that will meet every 6 months to review the trial and assess severe adverse events. The DSMB will include at least one epidemiologist, a statistician, and a clinician/social scientist, and half of the DSMB will comprise experts that have lived or worked in the local context. An independent, external study monitor will also conduct audits through the duration of the study. Protocol changes will be reviewed and approved by all ethics and regulatory boards. Additionally, any relevant changes will be updated on the trial registration at clinicaltrials.gov.

## Dissemination

The results of this study are intended to guide decisions related to the implementation and scale-up of MMS in pregnancy. Thus, we will be purposeful about consistent stakeholder engagement before, during, and after the clinical trial. We will disseminate the findings of our study through the publication of key research findings in peer-reviewed journals with suitable audiences. Authorship for trial publications will be determined by consensus of the principal investigators, with consideration for equity, fairness, and opportunity among those who have contributed to the study, including data collection, curation, and analysis*.* We will present selected results at conferences, research seminars, and scientific meetings with a focus on nutrition in the global health context. We will also communicate the findings back to the study participants and local communities to both acknowledge their contribution and share knowledge for capacity-building purposes. We will also disseminate our findings to government and non-government stakeholders. The audiences for our dissemination include study participants and local communities, local research partners, international researchers and academic partners, and public health and nutrition practitioners.

## Discussion

The benefits of MMS over IFA in improving birth outcomes are clear based on nearly two dozen randomized trials [18]. Many programs and countries are currently considering, or actively transitioning, from using IFA to using MMS during antenatal care, and the United Nations Children’s Fund (UNICEF) recently released *An Acceleration Plan to Prevent Malnutrition and Anaemia during Pregnancy* touting MMS as one of the five interventions that should be included in a package of essential services during pregnancy [30] . While this study is intended to inform policy and antenatal care recommendations in contexts where anemia in pregnancy is an important public health problem and the use of IFA with 60 mg of iron is standard of care, it will also inform programs currently using MMS. Ultimately, the results of this study will inform the optimal formulation of MMS.

## Supplementary Information

Additional file 1. Standard Protocol Items: Recommendations for Interventional Trials (SPIRIT) 2013 Checklist.

Additional file 2. Diagram of the regimen blister packs.

## Data Availability

The datasets used and/or analyzed during the current study may be made available from the corresponding author on reasonable request and approval from applicable Institutional Review Boards.

## Abbreviations

CI: Confidence interval
DHS: Demographic and Health Survey
DSMB: Data and Safety Monitoring Board
Hb: Hemoglobin
HDP: Hypertensive disorder of pregnancy
IFA: Iron-folic acid
IRB: Institutional Review Board
IU: International unit
LMICs: Low- and middle-income countries
MUAC: Mid-upper arm circumference
RR: Relative risk
SPIRIT: Standard Protocol Items: Recommendations for Interventional Trials
TAG: Technical Advisory Group
UNIMMAP: United Nations International Multiple Micronutrient Antenatal Preparation
WHO: World Health Organization
